# External Validation of a Multimodal Model for Predicting Outcomes in Preterm Newborns

**DOI:** 10.1001/jamanetworkopen.2025.23029

**Published:** 2025-07-31

**Authors:** Laura Routier, Sarah Touati, Ghida Ghostine-Ramadan, Fabrice Wallois, Laurent Querne, Emilie Bourel-Ponchel

**Affiliations:** 1 INSERM UMR 1105, Research Group on Multimodal Analysis of Brain Function, University of Picardie Jules Verne, Amiens, France; 2 Department of Pediatric Neurophysiology, Amiens-Picardie University Hospital, Amiens, France; 3 Neonatal Intensive Care Unit, Amiens-Picardie University Hospital, Amiens, France; 4 Department of Pediatric Neurology, Amiens-Picardie University Hospital, Amiens, France

## Abstract

**Importance:**

Predicting outcomes in preterm newborns is crucial for guiding therapeutic decisions and personalized rehabilitation. Prognostic models are a promising approach but rigorous validation is required to ensure their reliability and generalizability.

**Objective:**

To provide external validation of the PRETERM-POM model, a multimodal prognostic model developed for predicting outcomes in extremely preterm newborns at age 2 years.

**Design, Setting, and Participants:**

In this prognostic study conducted at Amiens-Picardie University Hospital (France), the original parameters of the model, developed through multivariate analysis and decision-tree algorithms, were applied to a temporal validation group of preterm newborns born between January 2018 and January 2021 that did not overlap with the development population. The study included preterm newborns born at 23 to 28 weeks of gestational age who had undergone cranial ultrasound and conventional electroencephalogram within the first 14 days after delivery and follow-up until a corrected age of 2 years.

**Main Outcomes and Measures:**

Neurodevelopment, assessed with the Denver Developmental Screening Test-II, was classified as favorable (no severe neurodevelopmental impairment [NDI]) or adverse (death or severe NDI). The area under the curve (AUC) for decision-tree classifications of adverse vs favorable outcomes was determined for the validation group and compared with the model development group using the DeLong test. Calibration and model fit were assessed using calibration-in-the-large, calibration curves, the Hosmer-Lemeshow test, and Brier score.

**Results:**

For the validation group (104 participants; median [IQR] gestational age at delivery, 26.3 [25.4-27.7] weeks; 46 [44.2%] male participants), the model achieved an AUC of 85.9% (95% CI, 79.0%-92.8%) for predicting outcomes, closely mirroring the performance observed in the development cohort. The model fit was good although it tended to underestimate the risk of adverse outcomes.

**Conclusions and Relevance:**

In this external validation study, the high performance of the model for predicting NDI in preterm newborns was confirmed. This multimodal approach, with a transparent display of risk factor contributions, enhances the potential of this model for clinical applications guiding timely management and decision-making.

## Key Points

**Question:**

Can a multimodal prognostic model predict neurodevelopmental outcomes reliably in extremely preterm newborns?

**Findings:**

In this prognostic study with 104 participants, the model had a performance similar to that for the model development cohort, with an area under the curve of 85.9% for predicting adverse vs favorable outcomes, and indicated a good fit of the model through calibration.

**Meaning:**

These findings suggest this multimodal model constitutes a robust, highly explainable approach for predicting neurodevelopmental outcomes in preterm newborns, providing valuable prognostic information to guide management decisions within the first 14 days after birth.

## Teaser

This prognostic study evaluates the performance of a multimodal prognostic model developed for predicting outcomes in extremely preterm newborns at 2 years of age.

## Introduction

Despite great improvements in neonatal resuscitation, preterm newborns remain at high risk of death and neurodevelopmental impairment (NDI). Early outcome prediction remains challenging. Clinicians lack prognostic tools to guide therapeutic decisions, provide parents with critically important information, and implement early personalized rehabilitation strategies to reduce neurological deficiencies and disabilities. Early stratification of newborns based on neurodevelopmental risk is also required for the development of new neuroprotective strategies and evaluations of their efficacy.

We recently developed a multimodal prognostic model that yielded promising results for predicting 2-year outcomes in extremely preterm newborns.^1^ Its distinctive feature lies in the integration of brain function and structure data alongside clinical and biological variables, all captured within the first 14 days after delivery. Using a data-learning procedure combining multiple factor analysis (MFA) and decision-tree classification, we achieved a transparent model displaying the respective contributions of each risk factor to the prognosis. We demonstrated and internally validated the high prognostic performance of this model with an area under the curve (AUC) of about 92% for predicting favorable vs adverse outcomes.^1^ Thus, we highlighted the superiority of this innovative strategy for early neurodevelopmental risk stratification compared with models that rely on a more limited set of prognostic indicators.^2,3,4,5^

External validation is crucial for ensuring the reliability, reproducibility and generalizability of predictive models before their clinical application in personalized medicine; however, such validation remains insufficiently performed.^6,7,8,9^ This study aimed to externally validate this multimodal model by rigorously testing its prognostic performance on a nonoverlapping population and by assessing its calibration.

## Methods

This prognostic study was approved by the French National Commission for Data Protection and Liberties and followed the Transparent Reporting of a Multivariable Prediction Model for Individual Prognosis or Diagnosis (TRIPOD) reporting guideline.^10^ In accordance with French legislation, the child's parents or legal representatives were informed about the study. Their consent was not directly required, but they could object to their child's inclusion at any time by expressing their refusal, either verbally or in writing.

### Original Multimodal Prognostic Model

The original PRETERM-POM (for Predicting Outcome with a Multimodal Model in Preterm infants) model has been described in detail elsewhere.^1^ The study design for the development group is available in eMethods 1 in Supplement 1.

#### Development Group

Briefly, the original model was developed using data from 109 preterm infants born at 23 to 28 weeks’ gestational age and admitted to the neonatal intensive care unit (NICU) at Amiens-Picardie University Hospital (France) between January 1, 2013, and January 1, 2018. Outcome was assessed at 2 years’ corrected age using the Denver Developmental Screening Test-II (DDST-II).^11^

#### Data Selection

The risk factors were collected during the first 14 postnatal days and classified into 4 categories: (1) perinatal, (2) postnatal, (3) brain structure (from cranial ultrasound [cUS]), and (4) brain function (from conventional electroencephalogram [cEEG]). Factors significantly associated with outcome in univariate analysis were used to build the model. The selected variables were: (1) Clinical Risk Index for Babies-II (CRIB-II) score^12^; (2) necrotizing enterocolitis,^13^ hemodynamic disorders, hypoxic-respiratory failure, patent ductus arteriosus and nonfatal cardiac arrest; (3) intraventricular hemorrhage grade^14^; and (4) cEEG richness and lability^15,16,17,18^; frontal, temporal, and occipital theta activities in coalescence with a slow wave^15,16,17,18^; delta-brush wave^15,16,17,18^; and negative central activity^15,19^ (for the definitions of these criteria, see eMethods 2 in Supplement 1, Routier et al^1^).

#### Model Development

The model was built by applying MFA to the 4 categories of selected variables. MFA is an extension of principal component analysis, in which the initial set of variables is structured into weighted groups (groups 1, 2, 3, and 4 in this study). MFA decomposes the global variance of the original variable set into new linear orthogonal variables (principal axes [PAs]), capturing the maximum amount of the variance between participants with a high degree of explainability (each original variable is associated with each PA via a specific contribution coefficient; see eMethods 3 in Supplement 1). For each PA, the contribution coefficients of all the original variables are summed (PA equations in eMethods 4 in Supplement 1).

A classification analysis by regression tree (CART) was then applied to the first 2 PAs generated by the MFA to define optimal cutoff points (nodes) separating adverse and favorable outcomes. CART identified 2 nodes (node-1 = +0.31 on PA1; node-2 = −0.89 on PA2), which delineated 3 distinct leaves in the PA1-PA2 plane, with the risk of an adverse outcome increasing from leaf-1 to leaf-3. In the development group, the model yielded an AUC of 91.7% (95% CI, 86.4%-97.0%) for predicting favorable vs adverse outcomes.

### External Validation, Population, and Model Transposition

#### Validation Group

For external validation, we applied a temporal strategy in a nonoverlapping population (study design for the validation group available in eMethods 1 in Supplement 1). The infants of the validation group, born between January 2, 2018, and January 1, 2021, and admitted to the NICU at Amiens-Picardie University Hospital, were included retrospectively. The inclusion and exclusion criteria were identical to those used in the development group^1^: infants born at 23 to 28 weeks’ gestational age who had undergone cEEG and cUS in the first 2 postnatal weeks with no genetic disease or major congenital malformation were included.

#### Outcome Assessment

As in the development group, neurodevelopmental outcome at 2 years’ corrected age was assessed with the DDST-II. Adverse outcomes were defined as death before 2 years’ corrected age or severe NDI, characterized by achieving less than 50% of age-appropriate milestones on the DDST-II. Favorable outcomes were defined as survival with achievement of more than 50% of DDST-II milestones.

#### Data Collection

All variables initially considered for the model development were collected. However, only those retained in the original model were considered for the external validation analysis.

#### Model Parameter Transposition

The PA equations derived from the original model were used to calculate the PA1 and PA2 coordinates for each newborn in the validation group, based on their risk factors (eMethods 4 in Supplement 1).

### Statistical Analysis

#### Group Comparison

The validation and development groups were compared for risk factors and outcomes. Univariate pairwise comparisons were performed with χ^2^ tests (or Fisher exact tests if χ^2^ tests were inappropriate) for binary variables, and *t* tests (or Mann-Whitney *U* tests if *t* tests were inappropriate) for quantitative variables. Depending on the statistical test used, the data are reported as relative risk (RR) with 95% CI, mean and SD, or median and IQR. The type I error for multiple comparisons was controlled by Benjamini-Hochberg (BH) correction (k = 20; *P* < .05). The AUC for predicting adverse vs favorable outcomes was calculated in the validation group and compared with that of the development group using the DeLong test.

#### Calibration and Fitting

Calibration-in-the-large was assessed and the calibration curve was generated^20^ to evaluate how well the original model accurately predicted favorable and adverse outcomes in the validation group. Model goodness-of-fit was evaluated using the Hosmer-Lemeshow test and the Brier score.

Statistical analyses were performed using R version 4.3.2 (R Project for Statistical Computing) (eMethods 5 in Supplement 1). *P* values less than .05 were considered statistically significant (all tests were 2-sided).

## Results

### Characteristics of the Validation Group

One hundred seventy-three preterm newborns born between 23 and 28 weeks’ gestational age were admitted to the NICU from 2018 to 2021, and 104 of these infants met the inclusion criteria. The median (IQR) gestational age at delivery was 26.3 (25.4-27.7) weeks, and 46 were male (44.2%). Risk factors of the validation group are summarized in the Table.^13,14,15,16,17,18^

**Table.** Risk Factor Scores and Statistical Pairwise Comparisons Between the Development and Validation Groups

Risk factor	Scoring	Participants, No.^a^		RR (95% CI)	*P* value^b^
		Development (N = 109)	Validation (N = 104)		
Perinatal risk factors					
CRIB-II,^12^ median (IQR)^c^	0 to 27	11 (10 to 14)	12 (10 to 13)	NA	.94^d^
Gestational age, median (IQR), wk		26.3 (25.1 to 27.3)	26.3 (25.4 to 27.7)	NA	.75^d^
Birth weight, median (IQR), g		800 (650 to 910)	795 (667 to 921)	NA	.79^d^
Body temperature on admission to the NICU, median (IQR), °C		36.1 (35.6 to 36.7)	36.0 (35.4 to 36.5)	NA	.28^d^
Base excess on admission to the NICU, median (IQR), mmol/L		−5.8 (−3.2 to −8.4)	−4.6 (−2.6 to −6.6)	NA	.11^d^
Sex (male:female) ratio		58:51	46:58	0.83 (0.63-1.1)	.47^d^
Postnatal morbidity (0-14 d)					
Necrotizing enterocolitis (Bell classification)^13^	0 to 3	Grade 0 and 1: 50 (41 and 9, respectively); grade 2 and 3: 59 (50 and 9, respectively)	Grade 0 and 1: 50 (28 and 22, respectively); grade 2 and 3: 54 (48 and 6, respectively)	0.96 (0.74-1.24)	.79^e^
Hypoxemic respiratory failure, (absent = 0; present = 1)	0 to 1	Absent: 65; present: 44	Absent: 68; present: 36	0.86 (0.60-1.22)	.66^e^
Hemodynamic disorders, (absent = 0; present = 1)	0 to 1	Absent: 41; present: 68	Absent: 49; present: 55	0.85 (0.67-1.07)	.46^e^
Nonfatal cardiac arrest, (absent = 0; present = 1)	0 to 1	Absent: 99; present: 10	Absent: 92; present: 12	1.26 (0.57-2.79)	.75^e^
Hemodynamically significant patent ductus arteriosus, (absent = 0; present = 1)	0 to 1	Absent: 42; present: 67	Absent: 33; present: 71	1.11 (0.91-1.35)	.66^e^
Structural brain injury (cUS) (0-14 d)					
Intraventricular hemorrhage (Papile classification),^14^ (grade 0 = 0; grade 1 = 1; grade 2 = 2; grade 3 = 3; grade 4 = 4)	0 to 4	Grade 0, 1 and 2: 65 (33, 14 and 18, respectively); grade 3 and 4: 44 (22 and 22, respectively)	Grade 0, 1 and 2: 72 (26, 21 and 25, respectively); grade 3 and 4: 32 (15, 17, respectively)	0.76 (0.53-1.10)	.46^e^
Brain function risk factors (cEEG) (0-14 d)^15,16,17,18,19^					
Lability (normal = 0; abnormal = 1)	0 to 1	Normal 92; abnormal: 17	Normal: 98; abnormal: 6	0.37 (0.15-0.90)	.14^e^
Richness (normal = 0; abnormal = 1)	0 to 1	Normal: 88; abnormal: 21	Normal: 82; abnormal: 22	1.10 (0.64-1.87)	.79^e^
Theta frontal activity coalescing with a slow wave, (normal = 0; disorganized = 1; absent = 2)	0 to 2	Normal: 35; disorganized and absent: 74 (57 and 17, respectively)	Normal: 28; disorganized and absent: 76 (66 and 10, respectively)	1.08 (0.90-1.28)	.66^e^
Theta temporal activity coalescing with a slow wave, (normal = 0; insufficient occurrence = 1; disorganized = 2; absent = 3)	0 to 3	Normal: 35; insufficient, disorganized and absent: 74 (16, 43 and 15, respectively)	Normal: 48; insufficient, disorganized and absent: 56 (1, 33 and 22, respectively)	0.79 (0.64-0.99)	.18^e^
Theta occipital activity coalescing with a slow wave, (normal = 0; disorganized = 1; absent = 2)	0 to 2	Normal: 50; disorganized and absent: 59 (44 and 15, respectively)	Normal: 43; disorganized and absent: 61 (52 and 9, respectively)	1.08 (0.86-1.37)	.73^e^
Delta brush wave, (normal = 0; insufficient occurrence = 1; disorganized = 2; absent = 3)	0 to 3	Normal: 55; insufficient, disorganized and absent: 54 (9, 34 and 11, respectively)	Normal: 74; insufficient, disorganized and absent: 30 (6, 11 and 13, respectively)	0.58 (0.41-0.83)	.04^e^
Negative central activity, (absent = 0; present = 1)	0 to 1	Absent: 72; present: 37	Absent: 63; present: 41	1.16 (0.82-1.65)	.66^e^
Outcome (2 y), per DDST-II^11^					
Favorable (no and moderate NDI) = 0; adverse (severe NDI and death) = 1	0 to 1	Favorable: 52; adverse: 57	Favorable: 44; adverse: 60	1.10 (0.86-1.41)	.66^e^

Abbreviations: cEEG, conventional electroencephalogram; CRIB-II, Clinical Risk Index for Babies-II; cUS, cranial ultrasonography; DDST-II, Denver Developmental Screening Test; NA, not applicable; NICU, neonatal intensive care unit; RR, relative risk with validation group as the numerator and development group as the denominator.

^a^ There were no missing data in either group.

^b^ All *P* values are reported after Benjamini-Hochberg correction (k = 20; *P* < .05).

^c^ The CRIB-II score was calculated from the following indicators: gestational age at birth, birth weight, sex, base excess, and body temperature on admission to the NICU.

^d^ Mann-Whitney *U* test.

^e^ χ^2^ Test.

Forty-four of the 104 newborns (42.3%) had a favorable outcome. The remaining 60 newborns (57.7%) had an adverse outcome, with 9 (8.7%) experiencing severe NDI and 51 (49.0%) dying in the NICU.

### Comparison of the Validation and Development Groups

The only risk factor that differed significantly between the development and validation groups was delta-brush wave (RR, 0.58; 95% CI, 0.41-0.83; BH corrected *P* = .04) (Table). The rate of adverse outcomes was slightly higher in the validation group (60 of 104 newborns [57.7%]) than in the development group (57 of 109 newborns [52.3%]), but this difference was not significant (RR, 1.10; 95% CI, 0.86-1.41; BH corrected *P* = .66).

### Prognostic Performance and Calibration of the Model

#### MFA and CART classification

**Figure 1.**
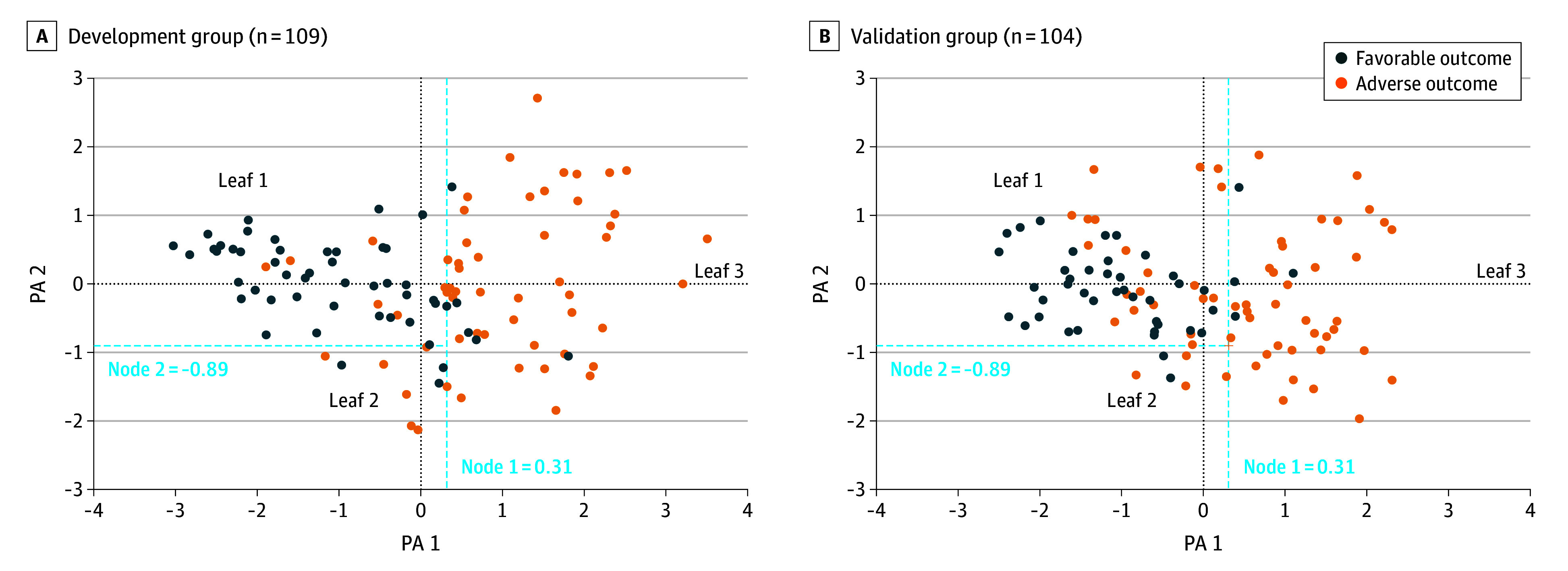
Projections on Principal Axes (PA) 1 and PA2 of Newborns From the Development and Validation Groups A, Coordinates of the 109 development-group newborns on the PA1 to PA2 plane of the multiple factor analysis (MFA) determining their assignment to leaf-1 (PA1 ≤ 0.31, PA2 ≥ −0.89 [classification and regression tree node-1 and -2, respectively]), leaf-2 (PA1 ≤ 0.31, PA2 < −0.89), or leaf-3 (PA1 > 0.31). B, The original multimodal model was applied to the 104 newborns in the validation group to calculate their coordinates on the MFA PA1-PA2 plane and determine their location in leaf-1, leaf-2, or leaf-3 based on the same nodes (node-1 = 0.31 and node-2 = −0.89). The newborns of the development group were distributed unevenly between outcomes, with the risk of adverse outcomes increasing from leaf-1 (12.0%) to leaf-2 and leaf-3 (66.7% and 92.0%, respectively). For the external validation group, the risk of an adverse outcome increased in a similar manner from leaf-1 (34.5%) to leaf-2 and leaf-3 (66.7% and 90.0%, respectively).

CART analysis classified the preterm newborns of the validation group into 3 leaves based on their coordinates calculated from the PA equations and based on the 2 nodes defined in the model development (node-1 = 0.31 on PA1; node-2 = −0.89 on PA2). As observed in the development group (Figure 1A), newborns in the validation group were unevenly distributed across the leaves, with the risk of adverse outcome increasing from leaf-1 (20 of 58 newborns [34.5%]) to leaf-2 and leaf-3 (4 of 6 newborns [66.7%] and 36 of 40 newborns [90.0%], respectively) (Figure 1B).

#### Prognostic Performance

**Figure 2.**
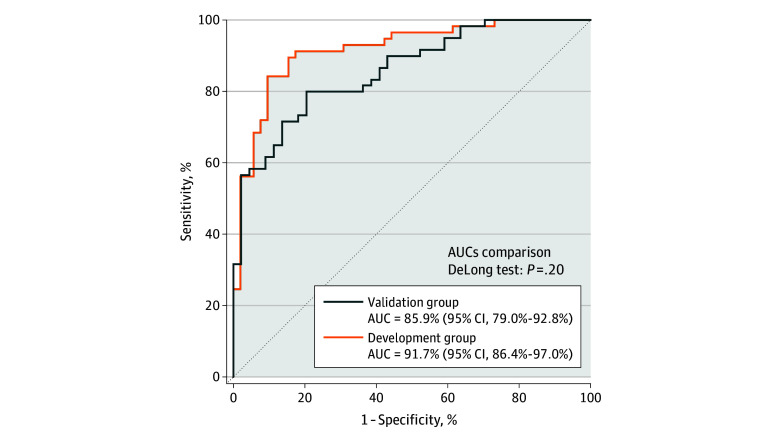
Performance of the Multimodal Prognostic Model in the Development and Validation Groups AUC indicates area under the curve.

The AUC for outcome prediction in the validation group was 85.9% (95% CI, 79.0%-92.8%), a performance not significantly different (*P* = .20) from that of the development group (91.7%; 95% CI, 86.4%-97.0%) (Figure 2). The positive and negative predictive values were 87.0% (95% CI, 74.3%-93.9%) and 65.5% (95% CI, 52.7%-76.4%), respectively.

#### Calibration and Fitting

**Figure 3.**
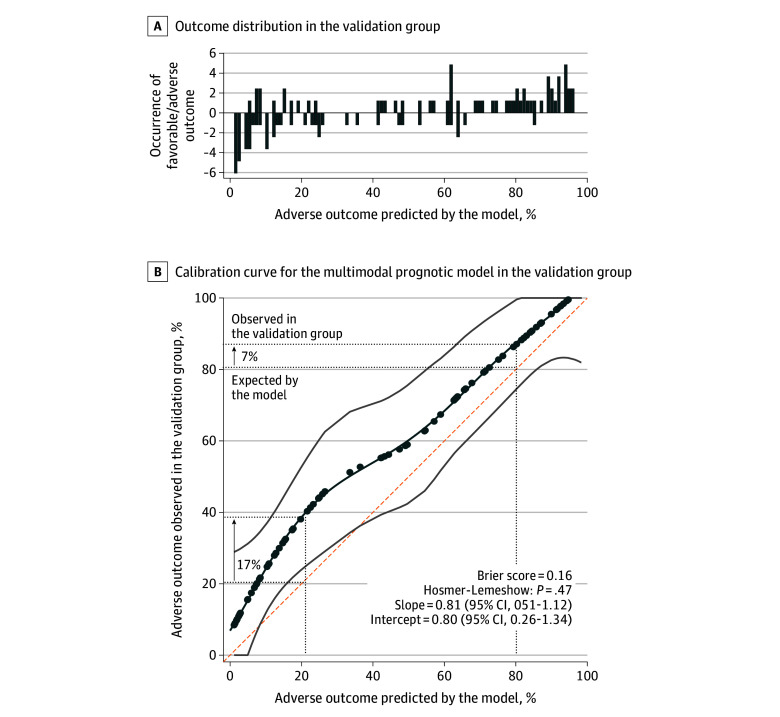
Calibration Curve for the Multimodal Prognostic Model in the Validation Group A, The vertical bars above the curve show the distribution of newborns along the horizontal axis according to their outcomes (favorable if ≤−1, adverse if ≥1) with the bars indicating their number. B, The calibration curve is represented as a dark blue line, and its 95% CI for the validation group is indicated by the gray band. The orange line (intercept = 0, slope = 1) represents the prediction of adverse outcomes in the development group calculated by the model. The circles indicate the positions of newborns from the validation group on the calibration curve graph. The slope of the calibration curve was less than 1, indicating that the risk of underestimation bias was more pronounced when the predicted risk of an adverse outcome was low (left side of the graph) than when this risk was high (right side). For example, when the model predicted a 20% probability of an adverse outcome, the observed proportion in the validation group was 37% (difference of 17%). In contrast, when the model predicted an 80% probability of an adverse outcome, the observed proportion was 87% (difference of 7%).

Calibration-in-the-large resulted in an intercept of 0.80 and a slope of 0.81, indicating that the model was well-calibrated when applied to the validation group, although it slightly underestimated the risk of an adverse outcome, particularly for the lowest predicted probabilities (Figure 3). As expected, the Hosmer-Lemeshow *P *value was not significant and the Brier-score of 0.16 remained lower than the value expected by chance (0.25), indicating a satisfactory fit of the calibration curve.

## Discussion

Our multimodal model demonstrated high prognostic performance in predicting 2-year outcomes for extremely preterm newborns. Similar AUCs were obtained for the development (91.7%; 95% CI, 86.4%-97.0%) and validation groups (85.9%; 95% CI, 79.0%-92.8%), with effective control of model overfitting. Thus, the performance of this model is comparable with that of the best models published to date.^2,4,5,21^ The model also has several key advantages absent from previous models: rigorous internal and external validation processes, early collection of risk factors, and clear explainability of the contributions of these risk factors to outcome prediction.

Recent advances in statistical and machine-learning tools have led to the development of numerous prediction models for enhancing personalized medicine, individualized decision-making, and risk stratification. However, to ensure the reliability, reproducibility, and generalizability of these models before their clinical application, a rigorous, standardized framework must be followed. This framework includes 3 critical stages: (1) model development and internal validation, (2) external validation and calibration, and (3) assessment of clinical impact.^6,7,8,9,10^

Despite the importance of these validation processes, they remain underused in current research practices. External validation is reported in less than 7% of model studies, and calibration often receives insufficient attention before clinical deployment, highlighting significant gaps in methods.^7,9^ This failure to follow the necessary procedures substantially increases the risk of overfitting models to the development dataset. For example, Li et al^5^ reported that a support vector machine algorithm achieved an impressive AUC of 98% during development, but its performance declined significantly, by 30%, at the validation phase, highlighting the critical need for independent external validation.^5,6,7^

We adhered closely to established methodological standards for our model development and validation, adopting a temporal approach for external validation. Our multimodal model displayed a slight, but expected decrease in prognostic performance in the validation group compared with the development cohort (6% decrease in AUC), which was not statistically significant. Calibration metrics confirmed that overfitting was effectively controlled with only a tendency to underestimate the risk of adverse outcomes in newborns with few risk factors. This underestimation may be explained by the limited data collection window, restricted to the 14 postnatal days, which excludes information on later-emerging complications. Importantly, newborns remain vulnerable throughout the neonatal period to developing additional morbidities that can negatively affect their long-term neurodevelopment. Nevertheless, this does not undermine the model’s primary strength, its ability to accurately predict outcomes using data from the first 2 postnatal weeks. Thus, the multimodal model addresses a critical clinical need by supporting timely management and decision-making in neonatal intensive care.

The inclusion of EEG and cUS variables improved the prognostic performance of the model, which is superior to that of early clinical and biological perinatal prediction models, such as CRIB-II score.^1^ Despite the limited data-collection window, incorporating these additional variables improved prognostic accuracy, enabling our model to outperform other models based on additional risk factors collected up to 3 to 6 months after birth.^2,4,22^ This early identification of the risk of adverse outcomes provides critical added value for the timely implementation of targeted rehabilitation strategies from the earliest postnatal days, leveraging neural network plasticity present during this critical period. Early risk stratification is also essential for the development of innovative therapeutic interventions and neuroprotective strategies.

Our multimodal model was specifically designed to optimize prognostic performance while minimizing overfitting. Unlike conventional approaches, MFA is outcome-blind, meaning that outcomes data are not incorporated during model construction; the model is derived solely from risk factors. This approach reduces the risk of overfitting and enhances generalizability to independent cohorts (see eMethods 6 in Supplement 1 for MFA performance in comparison with more conventional methods). Moreover, in contrast to many models based on data-driven methods that often lack transparency, our model is distinguished by a high level of explainability and a transparent methodology, providing clear insight into the contribution of each risk factor to the predicted outcome. Developed in accordance with TRIPOD guidelines, our model (1) provides straightforward equations that clarify the impact of each variable and (2) reveals its limitations, quantified through calibration analysis, which should promote adoption by health care practitioners. In a field in which black-box algorithms can impede external validation and clinical adoption, the transparency of our model ensures reliability and clarity—key characteristics for informed decision-making and enhancing patient outcomes.^4,7,10,23^

Ideally, our multimodal model should undergo validation in a prospective multicenter cohort in similar health care environments. Such validation is crucial to improve the predictive accuracy of the model in larger populations and to mitigate potential biases in participant selection, thereby ensuring broader applicability.

Future work will focus on simplifying the model while preserving its high prognostic performance to facilitate its integration into personalized medicine. This will include the development of a user-friendly clinical tool to assist health care professionals in stratifying neurodevelopmental risk in preterm newborns, providing risk estimates with clearly defined confidence levels to help to enhance and to personalize monitoring and care.

### Limitations

Several methodological limitations in the validation of the model warrant discussion. First, the adoption of a temporal validation strategy rather than a geographic validation strategy could attract criticism.^10^ Some researchers, such as Ramspek et al,^7^ advocate for geographic validation to ensure the generalizability and applicability of predictive models. However, the relative merits of the various types of external validation strategy remain a matter of debate.^9^ Youssef et al^9^ argued against relying solely on external validation to assess a model’s generalizability, reliability, safety, or utility, particularly in the complex context of caring for extremely preterm newborns in the NICU, as operational factors vary widely across health care settings. They proposed ongoing local validation to ensure the long-term validity of predictive models, which would favor the use of a site-specific approach to validate predictive tools before their clinical implementation.^9^

Second, the sample size of the model study may appear modest relative to recent recommendations on validation cohort size^24^ and those of other prognostic studies in preterm children, particularly those focusing solely on perinatal risk factors. However, with a cohort of more than 200 participants (development and validation groups), this model includes the largest sample to date in which cEEG results, either alone or in combination with other risk factors, have been considered.

Third, the retrospective, single-center nature of our study introduces a potential selection bias. The validation group did not significantly differ from the development group in terms of perinatal and postnatal characteristics or long-term outcomes. As in our previous study,^1^ the validation group included newborns with severe neonatal morbidity and adverse outcomes, suggesting a potential bias toward enrolling infants with more severe conditions and/or significant loss to follow-up by the corrected age of 2 years, particularly among those with favorable outcomes. This limitation may have restricted our ability to capture the full spectrum of long-term developmental trajectories.^1^

Fourth, we acknowledge that incorporating brain function information into clinical practice remains challenging, notably due to limited access to EEG-trained technicians and neurophysiologists, which may hinder broader adoption of the model. However, our findings reinforced prior recommendations advocating for the integration of cEEG data into prognostic models.^25,26^ Expert societies and original researches also support the routine use of EEG in preterm neonates, especially during the first postnatal weeks.^26,27,28,29,30^ By demonstrating that EEG data significantly improves outcome prediction, our multimodal model provides strong support for greater investment in EEG, currently the only tool available for routine monitoring of brain function in preterm newborns. Broader EEG implementation could be facilitated through the development of local or regional EEG networks.^27^ Standardizing EEG acquisition and interpretation procedures and adopting automated tools could enhance both the widespread application of EEG and the clinical use of the model.

## Conclusions

This study validated the PRETERM-POM model for predicting 2-year outcomes in extremely preterm newborns, confirming its robust performance and highlighting several key strengths. This multimodal model incorporates a comprehensive range of variables, including crucial data on brain structure and function, enhancing both its predictive accuracy and clinical relevance. Our methodology enhances transparency, providing clinicians with a clear understanding of the contribution of each risk factor to prognosis, in accordance with best practice.^10^ Despite the challenge of controlling overfitting during model development, our external validation study demonstrated consistently high predictive performance across independent populations. While the model showed a tendency to underestimate risks in infants with few risk factors, it remains a promising tool for the early identification of infants at risk, facilitating early interventions. The continued refinement of predictive models such as ours should bolster their reliability and increase their impact on clinical decision-making, ultimately improving long-term outcomes in preterm infants.

## Notes

Supplement 1. **eMethods 1.** Study Design **eMethods 2.** Risk Factor Definition and Analyses **eMethods 3.** Variable Contributions to the Multiple Factor Analysis **eMethods 4.** Equations of the Principal Axes 1 (PA1) and 2 (PA2) Based on Development Group Data and Applied to the Validation Group **eMethods 5.** R Package References **eMethods 6.** Prognostic Performance in the Training and Validation Samples According to Different Methodologies Used to Construct Multimodal Models
eReferences

Supplement 2.
Data Sharing Statement
