# Error in Results Section

**DOI:** 10.1001/jamanetworkopen.2025.34645

**Published:** 2025-08-27

In the Original Investigation titled “External Validation of a Multimodal Model for Predicting Outcomes in Preterm Newborns,”^1^ published July 31, 2025, there was an error in the Results section. The text should have indicated that 20 out of 58 newborns in leaf-1 had an increased risk of adverse outcomes. This article has been corrected.^1^
