# Supplementary material for: External Validation of a Multimodal Model for Predicting Outcomes in Preterm Newborns
Source: JAMA Netw Open. 2025 Jul 31;8(7):e2523029. doi: 10.1001/jamanetworkopen.2025.23029 (PMC12314722; doi:10.1001/jamanetworkopen.2025.23029)
Supplement: Supplement 1. — eMethods 1. Study Design eMethods 2. Risk Factor Definition and Analyses eMethods 3. Variable Contributions to the Multiple Factor Analysis eMethods 4. Equations of the Principal Axes 1 (PA1) and 2 (PA2) Based on Development Group Data and Applied to the Validation Group eMethods 5. R Package References eMethods 6. Prognostic Performance in the Training and Validation Samples According to Different Methodologies Used to Construct Multimodal Models eReferences [file jamanetwopen-e2523029-s001.pdf]

Supplemental Online Content

Routier L, Touati S, Ghostine-Ramadan G, Wallois F, Querné L, Bourel-Ponchel E. External validation of the PRETERM-POM model for predicting outcomes in preterm newborns. *JAMA Netw Open*. 2025;8(7):e2523029. doi:10.1001/jamanetworkopen.2025.23029

eMethods 1. Study Design

eMethods 2. Risk-Factor Definition and Analyses

eMethods 3. Variable Contributions to the Multiple Factor Analysis

eMethods 4. Equations of the Principal Axes 1 (PA1) and 2 (PA2) Based on Development Group Data and Applied to the Validation Group

eMethods 5. R Package References

eMethods 6. Prognostic Performance in the Training and Validation Samples According to Different Methodologies Used to Construct Multimodal Models

eReferences

This supplemental material has been provided by the authors to give readers additional information about their work.

© 2025 Routier L et al. *JAMA Netw Open*.

eMethods 1. Study Design

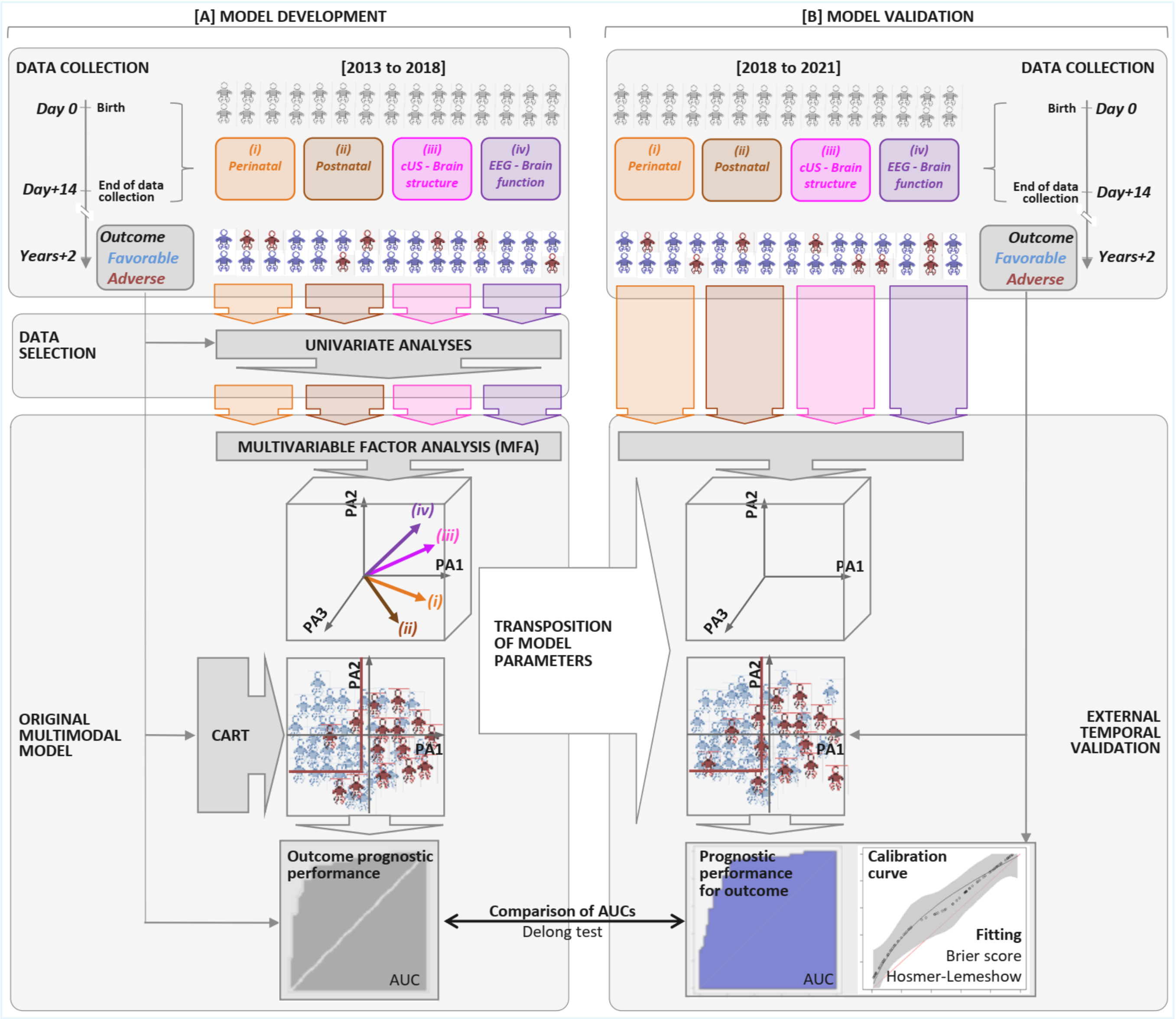

cUS: cranial ultrasound; EEG: electroencephalogram; MFA: multiple factor analysis; PA: principal axis; AUC: area under the curve; CART: classification and regression tree analysis.

eMethods 2. Risk-Factor Definition and Analyses

The variables of the PRETERM-POM model were collected and analyzed in accordance with the criteria used in the development study and detailed below[1].

Perinatal variables

The Clinical Risk Index for Babies-II (CRIB-II) is a score widely used by physicians to predict the initial risk of mortality on the basis of the following clinical and laboratory characteristics: gestational age, sex, birth weight, body temperature, and base excess on admission to the NICU[2].

Postnatal morbidity (in the 14 days after delivery)

• Bell's classification was used to assess necrotizing enterocolitis (NEC). NEC of grade 2 or above is considered to be a severe postnatal morbidity[3].

• Hypoxic respiratory failure (HRF) was defined as the presence of at least one of the following characteristics: $FiO_2 \geq 60\%$ or positive expiratory pressure (PEP) ≥ 6 cmH_2O for 2 hours, the use of high-frequency oscillatory ventilation or inhaled nitric oxide.

• Hemodynamic disorders (HD) requiring the use of vaso-active drugs were considered.

• Non-fatal cardiac arrest (CA) was defined as a lasting decrease in heart rate to below 60 beats/minute not resulting in death.

Brain structure risk factors (according to cranial ultrasound, cUS) (in the 14 days after delivery)

The presence/absence and severity of intraventricular hemorrhage (IVH) were scored according to Papile's classification[4].

0 = no IVH.

1 = IVH limited to the germinal matrix.

2 = IVH that fills less than 50% of the ventricles.

3 = IVH with extension into the dilated ventricle.

4 = IVH with parenchymal extension.

Function brain risk factors (conventional EEG (cEEG)) (in the 14 days after delivery)

All conventional EEG were interpreted and classified by two experienced neurophysiologists (LR and EBP) blind to clinical information other than postmenstrual age (PMA) at EEG recording and blind to outcome. The cEEG signal was analyzed by considering the general organization of electrical neural activities, the characteristics of specific maturational EEG features, and superimposed pathological features.

© 2025 Routier L et al. *JAMA Netw Open*.

General organization of the EEG

• Lability was defined as the presence of fluctuations between quiescent (discontinuous) and active periods (bursts of activity) and amplitude/frequency modulations of the features of the EEG[5–7]. Lability was scored as normal (0) or absent (1).

• Richness was assessed in terms of the proportion and duration of quiescent periods (QPs) (interburst intervals) and bursts of activity, as a function of the PMA. Richness was considered to be normal if the QPs lasted less than 35 s and the bursts of activity lasted for more than 10 s for cEEG recordings acquired at 24-28 weeks of PMA (wPMA). For cEEG recordings acquired at 29-30 wPMA, richness was considered to be normal if the QPs lasted less than 20 s and the bursts of activity lasted for more than 20 s[7]. Richness was scored as normal (0) or insufficient (1).

Specific maturational features on EEG

The localization and morphology of maturational EEG features (occipital, temporal, and frontal theta activities coalescing with a slow wave (TOA-SW, TTA-SW, and TFA-SW, respectively), and delta brush waves) were analyzed. TTA-SW, TFA-SW and TOA-SW were characterized by bursts of sharp 4-7 Hz waves coalescing with a biphasic slow wave, with an amplitude of 100-400 µV, localized in the temporal, frontal, and occipital regions, respectively[5–8]. TTA-SW normally appear with a frequency of at least 1 per 2 min at 24-25 wPMA and 1 per min at 26-30 wPMA. Delta brush waves were defined as monophasic or biphasic slow waves (0.5-1 Hz) that were smooth or superimposed on fast rhythms (8-25 Hz), with an amplitude of 100-400 µV, and appeared in sequences of several seconds[5–8]. Disorganized theta activities coalescing with a slow wave were defined as a deformed pattern with at least one of the following criteria: amplitude ≥ 500 µV, fast activities < 4 Hz or >7 Hz, sharp theta activities, and the absence of a slow wave[9–11]. Delta brush waves were considered to be disorganized if they were deformed and met at least one of the following criteria: lack of smoothness, a wider base, frequency < 0.5 Hz, amplitude > 500 µV, a mechanical cogwheel appearance, or with fast activities invading the whole slow wave[9–11]. An insufficient occurrence of TTA-SW was defined as < 2/min at 24-26 wPMA and < 1/min at 27-30 wPMA. Delta brush waves were insufficient if they appeared singly or at a frequency below 1/min.

• For TOA-SW and TFA-SW, the scores ranged from 0 to 2 (normal=0; disorganized=1; absent=2).

• For TTA-SW and delta brush waves, the scores ranged from 0 to 3 (normal=0; insufficient occurrence=1; disorganized=2; absent=3).

Pathological features

Negative central activity (NCA) was defined as isolated or sequential activity with a negative theta polarity in central regions (EEG electrodes: C4-C3-Cz) and an amplitude greater than 25 µV[12]. NCA was scored as absent (0) or present (1).

© 2025 Routier L et al. *JAMA Netw Open*.

eMethods 3. Variable Contributions to the Multiple Factor Analysis

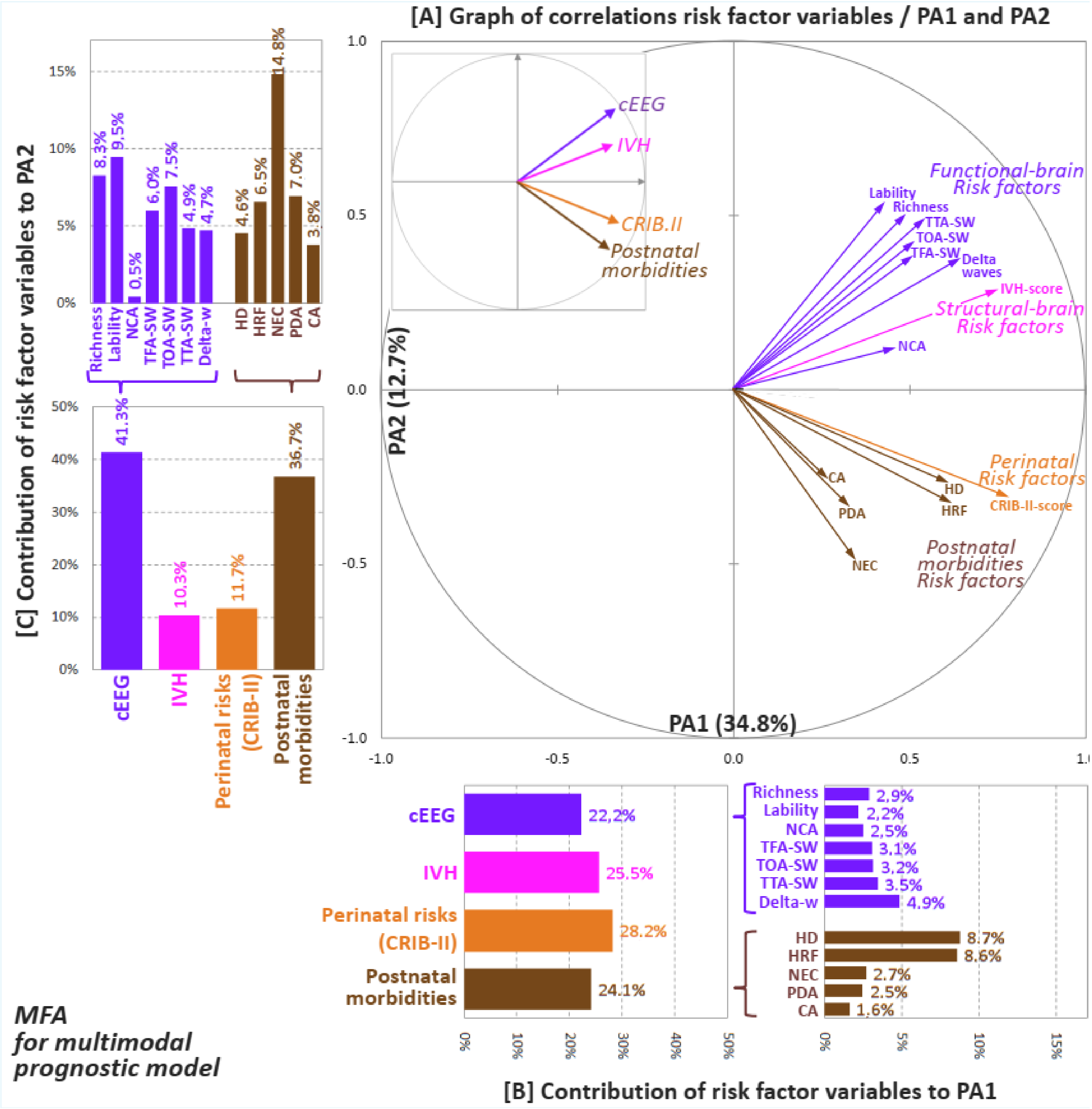

[A] Graph of the correlations between the variables and the two first PAs from the Multiple Factor Analysis (MFA). The groups of variables defined in the MFA were the CRIB.II score for perinatal risk factors, HD, HRF, PDA, NEC and CA for postnatal morbidities, IVH grades for brain structure risk factors, and TOA-SW, TTA-SW, TFA-SW, delta-wave, NCA, lability and richness of cEEG for brain function risk factors. Length and direction of each vector determined the specific contribution of the original variable to Principal Axes (PAs). Vectors resulting from each group of risk-factors were represented in the inset. [B] and [C] Contributions of the four risk-factor categories to PA1 and PA2, respectively.

© 2025 Routier L et al. *JAMA Netw Open*.

CA: non-fatal cardiac arrest; cEEG: conventional EEG; CRIB-II: Clinical Risk Index for Babies-II; DBW: delta brush wave; HD: hemodynamic disorder; HRF: hypoxic respiratory failure; IVH: intraventricular haemorrhage; Lability and richness: cEEG lability and richness, respectively; NEC: necrotizing enterocolitis; PA: principal axis; PDA: patent ductus arteriosus; TOA-SW, TTA-SW and TFA-SW: occipital, temporal and frontal theta activities in coalescence with a slow wave, respectively; NCA: negative central activity.

eMethods 4. Equations of the Principal Axes 1 (PA1) and 2 (PA2) Based on Development Group Data and Applied to the Validation Group

PA1 = -4.248 +0.201×CRIB-II +0.424×HRF +0.434×HD +0.23×PDA +0.112×NEC +0.313×CA +0.331×IVH +0.211×lability +0.22×richness +0.13×TOA +0.089×TTA +0.134×TFA +0.103×DBW +0.171×NCA

PA2 = +1.345 -0.129×CRIB-II -0.371×HRF -0.314×HD -0.386×PDA -0.261×NEC -0.481×CA +0.210×IVH +0.435×lability +0.374×richness +0.179×TOA +0.131×TTA +0.169×TFA +0.101×DBW +0.073×NCA

CA: non-fatal cardiac arrest; CRIB-II: Clinical Risk Index for Babies-II; DBW: delta brush wave; HD: hemodynamic disorders; HRF: hypoxic respiratory failure; IVH: intraventricular haemorrhage; Lability and richness: cEEG lability and richness, respectively; NEC: necrotizing enterocolitis; PDA: patent ductus arteriosus; grade; TOA-SW, TTA-SW and TFA-SW: occipital, temporal and frontal theta activities in coalescence with a slow wave, respectively; NCA: negative central activity.

eMethods 5. R Package References

R software

R Core Team. R: a language and environment for statistical computing. R Foundation for Statistical Computing. <https://www.R-project.org/>

Multiple factor analysis (MFA)

Lê S, Josse J, Husson F. FactoMineR. An R Package for Multivariate Analysis. J Stat Softw. 2008;25:1-18. doi:10.18637/jss.v025.i01

Vaissie P, Monge A, Husson F. Factoshiny: Perform Factorial Analysis from 'FactoMineR' with a Shiny Application. R package version 2.5. 2023. https://CRAN.R-project.org/package=Factoshiny

Classification analysis by regression tree (CART)

© 2025 Routier L et al. *JAMA Netw Open*.

Hothorn T, Zeileis A. Partykit: A Modular Toolkit for Recursive Partytioning in R. J Mach Learn Res. 2015;16(118):, 3905-3909. https://jmlr.org/papers/v16/hothorn15a.html

Therneau T, Atkinson B. Rpart: Recursive Partitioning and Regression Trees. R package version 4.1.21. 2023. https://CRAN.R-project.org/package=rpart

Area under the curve (AUC)

Robin X, Turck N, Hainard A, Tiberti N, Lisacek F, Sanchez JC and Müller M. pROC: an open-source package for R and S+ to analyze and compare ROC curves. BMC Bioinformatics, 2011;12-77. doi: 10.1186/1471-2105-12-77.

Calibration

Van Calster B, Nieboer D, Vergouwe Y, De Cock B, Pencina MJ, Steyerberg EW. A calibration hierarchy for risk models was defined: from utopia tempirical data. J Clin Epidemiol. 2016;74:167-176. doi: 10.1016/j.jclinepi.2015.12.005.

De Cock B, Nieboer D, Van Calster B, Steyerberg EW, Vergouwe Y. The Calibration Curves package: assessing the agreement between observed outcomes and predictions. 0R package version 2.0.0. 2023. <https://cran.r-project.org/package=CalibrationCurves>

Lüdecke D, Ben-Shachar MS, Patil I, Waggoner P, Makowski5 D. Performance: An R Package for Assessment, Comparison and Testing of Statistical Models. J Open Source Soft. 2021;6(60):3139. doi: 10.21105/joss.03139

eMethods 6. Prognostic Performance in the Training and Validation Samples According to Different Methodologies Used to Construct Multimodal Models

	**A**<br>MFA + CART performed on all the 21 variables (before univariate analyses)	**B**<br>MFA + CART performed on the 12 variables selected by univariate analyses	**C**<br>Logistic-regression performed on the 12 variables selected by univariate analyses	**D**<br>Logistic-regression performed on CRIB.II, HD, NEC and IVH	**E**<br>CART alone performed on the 12 variables selected by univariate analyses
AUC for the training sample	89.7%	91.7%	92.0%	86.1%	90.5%
AUC for the validation sample	#	85.9%	72.8%	80.9%	68.7%
AUC loss for the validation sample	#	-5.8%	-19.2%	-5.2%	-21.8%

Prognostic performance in the training sample and the validation sample according to different methodologies used to construct the multimodal models: A-Multimodal model created by combining a multiple factor analysis (MFA) and a classification and regression tree analysis (CART) on all the 21 risk factors, B-multimodal model created by combining a multiple factor analysis and a CART on the 12 risk factors selected through univariate analyses, C–multimodal model created using logistic regression on the 12 risk factors selected through univariate analyses. D–multimodal model created using logistic regression on 4 risk factors, E–multimodal model created using CART applied directly to the 12

© 2025 Routier L et al. *JAMA Netw Open*.

risk factors selected through univariate analyses. AUC: area under the curve; CART: classification and regression tree analysis; CRIB.II: Clinical Risk Index for Babies-II; HD: hemodynamic disorder; IVH: Intravenricular hemorrhage; MFA: multiple factor analysis; NEC: necrotizing enterocolitis.

eReferences

1. Routier L, Querne L, Ghostine-Ramadan G, et al. Predicting the Neurodevelopmental Outcome in Extremely Preterm Newborns Using a Multimodal Prognostic Model Including Brain Function Information. *JAMA Netw Open*. 2023;6(3):e231590. doi:10.1001/jamanetworkopen.2023.1590

2. Parry G, Tucker J, Tarnow-Mordi W. UK Neonatal Staffing Study Collaborative Group. CRIB II: an update of the clinical risk index for babies score. *Lancet*. 2003;361(9371):1789-1791. doi:10.1016/S0140-6736(03)13397-1

3. Bell MJ, Ternberg JL, Feigin RD, et al. Neonatal necrotizing enterocolitis. Therapeutic decisions based upon clinical staging. *Ann Surg*. 1978;187(1):1-7. doi:10.1097/00000658-197801000-00001

4. Papile LA, Burstein J, Burstein R, Koffler H. Incidence and evolution of subependymal and intraventricular hemorrhage: A study of infants with birth weights less than 1,500 gm. *J Pediatr*. 1978;92(4):529-534. doi:10.1016/S0022-3476(78)80282-0

5. Lamblin MD, André M, Challamel MJ, et al. Électroencéphalographie du nouveau-né prématuré et à terme. Aspects maturatifs et glossaire. *Neurophysiol Clin*. 1999;29(2):123-219. doi:10.1016/S0987-7053(99)80051-3

6. André M, Lamblin MD, d'Allest AM, et al. Electroencephalography in premature and full-term infants. Developmental features and glossary. *Neurophysiol Clin*. 2010;40(2):59-124. doi:10.1016/j.neucli.2010.02.002

7. Bourel-Ponchel E, Gueden S, Hasaerts D, et al. Normal EEG during the neonatal period: maturational aspects from premature to full-term newborns. *Neurophysiol Clin*. 2021;51(1):61-88. doi:10.1016/j.neucli.2020.10.004

8. Wallois F, Routier L, Heberlé C, Mahmoudzadeh M, Bourel-Ponchel E, Moghimi S. Back to basics: the neuronal substrates and mechanisms that underlie the electroencephalogram in premature neonates. *Neurophysiol Clin*. 2021;51(1):5-33. doi:10.1016/j.neucli.2020.10.006

9. Watanabe K, Hayakawa F, Okumura A. Neonatal EEG: a powerful tool in the assessment of brain damage in preterm infants. *Brain Dev*. 1999;21(6):361–72. doi:10.1016/s0387-7604(99)00034-0

10. Tich SNT, d'Allest AM, Villepin AT de, et al. Pathological features of neonatal EEG in preterm babies born before 30 weeks of gestationnal age. *Neurophysiol Clin*. 2007;5(37):325-370. doi:10.1016/j.neucli.2007.10.001

11. Hayashi-Kurahashi N, Kidokoro H, Kubota T, et al. EEG for Predicting Early Neurodevelopment in Preterm Infants: An Observational Cohort Study. *Pediatrics*. 2012;130(4):e891-e897. doi:10.1542/peds.2012-1115

12. Routier L, Edalati M, Querné L, et al. Negative central activity in extremely preterm newborns: EEG characterization and relationship with brain injuries and neurodevelopmental outcome. *Clin Neurophysiol*. 2024;163:236-243. doi:10.1016/j.clinph.2024.04.006

© 2025 Routier L et al. *JAMA Netw Open*.
