# Supplementary material for: External Validation of a Multimodal Model for Predicting Outcomes in Preterm Newborns
Source: JAMA Netw Open. 2025 Jul 31;8(7):e2523029. doi: 10.1001/jamanetworkopen.2025.23029 (PMC12314722; doi:10.1001/jamanetworkopen.2025.23029)
Supplement: Supplement 2. — Data Sharing Statement [file jamanetwopen-e2523029-s002.pdf]

Data Sharing Statement

Routier. External Validation of the PRETERM-POM Model for Predicting Outcomes in Preterm Newborns. *JAMA Netw Open*. Published July 24, 2025.
doi:10.1001/jamanetworkopen.2025.23029

Data
Data available: No
